# Wear of Functionally Graded Coatings under Frictional Heating Conditions

**DOI:** 10.3390/nano12010142

**Published:** 2021-12-31

**Authors:** Vladimir B. Zelentsov, Polina A. Lapina, Boris I. Mitrin

**Affiliations:** Research and Education Center “Materials”, Don State Technical University, 344000 Rostov-on-Don, Russia; vbzelen@gmail.com (V.B.Z.); boris.mitrin@gmail.com (B.I.M.)

**Keywords:** functionally graded material, thermoelasticity, sliding contact, wear, heating from friction, thermoelastic instability

## Abstract

Multilayered and functionally graded coatings are extensively used for protection against wear of the working surfaces of mechanisms and machines subjected to sliding contact. The paper considers the problem of wear of a strip made of a functionally graded material, taking into account the heating of the sliding contact from friction. Wear is modeled by a moving strip along the surface of a hard abrasive in the form of a half-plane. With the help of the integral Laplace transform with respect to time, the solutions are constructed as convolutions from the law of the introduction of an abrasive into the strip and the original in the form of a contour integral of the inverse Laplace transform. The study of the integrands of contour quadratures in the complex plane allowed determination of the regions of stable solutions to the problem. Unstable solutions of the problem lead to the concept of thermoelastic instability of the contact with friction and formed regions of unstable solutions. The solutions obtained made it possible to determine a formula for the coefficient of functionally graded inhomogeneity of the coating material and to study its effect on the occurrence of thermoelastic instability of the contact taking friction into account, as well as on its main characteristics: temperature, displacement, stress and wear of the functionally graded material of the coating. The effects of the abrasive speed, contact stresses and temperature on wear of the coating with the functionally graded inhomogeneity of the material by the depth were investigated.

## 1. Introduction

Design of multilayered materials increased rapidly in the 1980–1990s, thus enabling the introduction of such materials with properties variable by depth in a number of industries such as construction, transport and other fields. Overall, their necessity in such fields was due to the increasing requirements for strength, wear and tear of details and parts. Recently, methods and technologies for the manufacturing of protective coatings from functionally graded materials (FGM) to micro- and nanometer technological levels have been developed: the centrifugal method [1,2,3,4,5,6,7], the technique of pulsed-laser deposition [8,9], the technique of magnetron sputtering [9], the plasma-spray technique [10,11], electrophoretic deposition and anodizing [12,13], micro-arc oxidation [14] and others [15].

FGM coatings are widely used to protect friction surfaces from wear. Generally, the tribological properties of FGM coatings are investigated by experimental methods, such as disc–pad [1,5], ball–disc [3,9,11], pin-on-disc [4,13,15], ring–pad [8], pin–plate [10] and other tests [7,14]. The coating wear as a result of testing is determined by examining the wear track with a microscope or a profilometer [1,2,3,9,10,13] or by weighing the sample before and after the test [4,5,6,7,8,11,15]. In this case, tests are carried out at one value of the experimental load or for a limited set (3–5 options).

The need to optimize the design of FGM coatings, to predict wear of the working surfaces of mechanisms and machines, to diagnose and prevent abnormal situations necessitates mathematical modeling of the wear process. In modern microelectronics, semiconductor materials are used in the form of thin plates with a thickness not exceeding 20–30 µm down to 100 nm, and the presence of a functionally graded inhomogeneity of the material (FGIM) by depth. The process of thinning such plates in practice is carried out by grinding, polishing and washing. Mathematical modeling of the grinding process of the workpiece material is associated with the elucidation of the degree of influence of the properties of the FGIM of the workpiece material on the grinding process; on the nature of the workpiece heating; on the possibility of the occurrence of thermoelastic instability of the sliding contact; on acceleration/deceleration the grinding process, etc.

A lot of research is devoted to mathematical modeling of the contact of bodies with FGM coatings [16,17,18,19,20,21,22,23,24,25,26]. However, insufficient attention has been paid to the problem of modeling of wear or grinding of FGM coatings. There has been little research conducted in this direction [27].

Today, when solving problems of material wear, Archard’s relations are most often used [28]. Dow and Burton were among the first investigators who used the Archard relation in the study of wear under conditions of heat release from friction [29], where the conditions for the occurrence of thermoelastic instability of blade sliding along the surface of a half-space were investigated using the method of small perturbations. Aleksandrov and Annakulova considered contact problems taking into account heat release from friction and wear of the coating [30], as well as the problem of mutual wear of coatings [31]. An attempt to develop a thermodynamic model to describe thermomechanical phenomena at a contact, taking into account friction and wear, was undertaken in [32,33]. Beginning with [34,35] a new direction of the development of the model of contact of two elastic bodies considering friction, wear and heat release, based on the principle of virtual energy and the basic laws of thermodynamics, has emerged. The finite element implementation of such a model in a two-dimensional statement was conducted in [36]. In [37,38,39,40,41] the integral Laplace transform was used with the solution in the form of functional series over the poles of the integrands of the contour quadratures of the inverse Laplace transform to solve contact thermoelasticity problems regarding wear. The solution method allows one to establish the parameters of the boundary of thermoelastic instability of a contact with a friction to study the properties of the obtained solutions. In [42], using the method of integral transforms, the contact problem of sliding an elastic coating over the surface of another coating with friction, wear and heat release from friction was reduced to solving a differential equation, and the conditions of thermoelastic stability of such a system were considered. Quasi-static and dynamic uncoupled contact problems of thermoelasticity regarding friction and wear of a rod were considered in [43]. The conditions for the thermoelastic instability emergence during mutual wear of surfaces made of different materials were considered in [44,45]. Due to the large number of parameters in the problems of thermofrictional contact and wear, one-dimensional quasi-static problems were often considered. The connectivity of the deformation fields and temperature in the listed works was neglected and the problems of uncoupled thermoelasticity were considered. The coupled problem of thermoelasticity on wear of a coating taking into account frictional heat release in a quasi-static formulation was considered in [46].

In the present paper, we consider a problem of wear of an elastic coating in the form of a strip made of FGM by a hard abrasive in the form of a half-plane, which slides along the strip at a constant speed, heating it due to friction. The influence of the FGIM of the strip on the process of wear (grinding), heating from friction, the conditions for the occurrence of thermoelastic instability of the sliding contact are investigated.

## 2. Problem Statement

**Figure 1:**
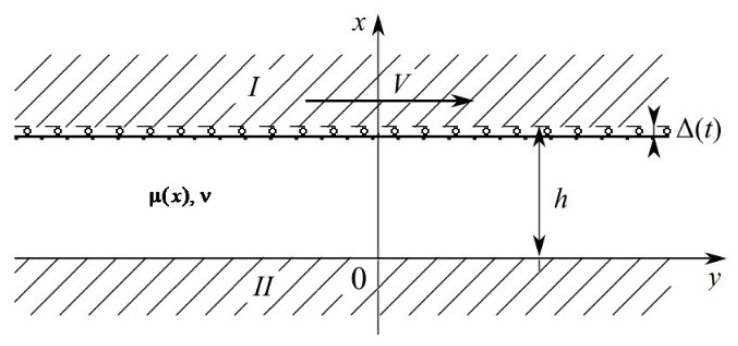
Scheme of the contact problem.

To investigate the effect of the FGIM of the coating on its wear we consider a quasi-static contact problem of sliding of a rigid heat-insulated abrasive, half-plane *I* (h≤x<∞), sliding with constant velocity V, over the upper surface (x=h) of the elastic thermally conductive coating with thickness h(0≤x≤h). The lower surface of the coating is perfectly bonded to a rigid substrate, half-plane *II* (−∞<x<0). The coating shear modulus μ(*x*) varies with depth 0≤x≤h. During abrasive (half-plane *I*) sliding, the coating wear takes place, which can also be thought of as the abrasive grinding of the coating surface. The frictional heat generated at the contact interface flows into the coating. From the initial time moment, the abrasive (half-plane *I*) slides along the y axis and deforms the upper (x=h) surface of the coating in the negative direction of the x axis according to the indentation law Δ(t). Until the initial time moment, the coating was at rest, and its temperature was equal to zero. The scheme of the contact problem is shown in Figure 1.

In the described problem formulation, the temperature, stresses and displacements distributions in the coating do not depend on the horizontal coordinate y and are functions only of the vertical coordinate x and time t [37,38,39,40,41]. In the case of a quasi-static formulation without body forces, the stress state of the coating is described by the differential equations of equilibrium

(1) $$\frac{\partial \sigma_{xx}}{\partial x}=0,\;\frac{\partial \sigma_{xy}}{\partial x}=0,\;\;0\leq x\leq h,\;\;t>0$$

where $\sigma_{xx}(x,t)$, $\sigma_{xy}(x,t)$ are the normal and shear components of the stresses in the coating.

The heat equation has the form

(2) $$\frac{\partial^{2}T}{\partial x^{2}}-\frac{1}{\kappa }\frac{\partial T}{\partial t}=0\;\;0\leq x\leq h,\;\;t>0$$

where T(x,t) is the coating temperature, κ is the thermal diffusivity.

The Duhamell–Neumann law takes place [47]

(3) $$\sigma_{xx}=\frac{2(1-\nu )}{1-2\nu }\mu (x)\left(\frac{\partial u}{\partial x}-\frac{1+\nu }{1-\nu }\alpha T\right),\;\sigma_{xy}=\mu (x)\frac{\partial w}{\partial x},$$

where u(x,t), w(x,t) are the vertical and horizontal components of the displacements in the coating, μ(x), ν, α are the shear modulus, Poisson’s ratio, coefficient of linear heat expansion of the coating material.

The differential equations of linear uncoupled elasticity are represented by the system of equilibrium Equation (1) and heat Equation (2), which together describe thermoelastic state of the coating. 

Boundary conditions for Equation (1) are as follows (t>0):

(4) $$u(h,t)=-\Delta (t)+u_{w}(t)$$

(5) $$\sigma_{xy}(h,t)=-f\sigma_{xx}(h,t)$$

(6) u(0,t)=0

(7) w(0,t)=0

where f is the coefficient of friction, $u_{w}(t)$ is the abrasive (half-plane *I*) displacement due to the coating wear. To find the solution, we use the abrasive wear model [23] in integral form

(8) $$u_{w}(t)=-fVK^{*}\int_{0}^{t}\sigma_{xx}(h,\tau )d\tau \;\;t>0$$

where $\sigma_{xx}(h,t)$ is the compressive normal stress on the contact interface, $K^{*}$ is the proportionality coefficient between the work of friction forces and the volume of removed material.

The boundary conditions for the heat Equation (2) are (t>0)

(9) $$K\frac{\partial T(h,t)}{\partial x}=Q(t)$$

(10) $$K\frac{\partial T(0,t)}{\partial x}=k\left(T(0,t)-T(0,0)\right)$$

where K is the thermal conductivity of the coating material, k is the heat transfer coefficient through the coating–substrate interface, $Q(t)=fV(-\sigma_{xx}(h,t))$ is the amount of frictional heat originatingfrom the contact interface [48]. From (9) it follows that all the heat at the contact is due to friction.

Initial conditions for displacements and temperature are equal to zero:

(11) u(x,0)=w(x,0)=T(x,0)=0, 0≤x≤h,

Thus, the solution of the considered quasi-static thermoelastic contact problem for the elastic FGM coating wear (or grinding) by a certain depth with a hard abrasive in the form of a half-plane, taking into account heating from friction, is derived through the solution of the initial boundary value problem, including the system of the differential equations of elasticity (1) and heat conduction (2) with boundary (4)–(10) and initial (11) conditions. Note that vertical displacements u(x,t), normal stresses $\sigma_{xx}(x,t)$ and temperature T(x,t) are found separately from the horizontal displacements w(x,t). The horizontal displacements w(x,t) are determined from (1), (5), (7) knowing the normal stresses’ distribution $\sigma_{xx}(h,t)$.

## 3. Exact Solution for Arbitrary µ(x)

We proceed to the solution of the formulated problem introducing the Laplace integral transform [49]

(12) $$T^{L}(x,p)=\int_{0}^{\infty }T(x,t)e^{-pt}dt,\;T(x,t)=\frac{1}{2\pi i}\int_{-i\infty +c}^{i\infty +c}T^{L}(x,p)e^{pt}dp\;\;\mathrm{Rep}<c,\;c>0$$

To obtain the temperature distribution T(x,t) in the coating, we apply the Laplace transform to heat Equation (2). As a result, the Laplace image $T^{L}(x,p)$ of the coating temperature has the form

(13) $$T^{L}(x,p)=A_{1}\mathrm{sh}\sqrt{\frac{p}{\kappa }}x+A_{2}\mathrm{ch}\sqrt{\frac{p}{\kappa }}x$$

where $A_{1}$ and $A_{2}$ are arbitrary constants.

The vertical displacements u(x,t) are determined from the first equilibrium Equation (1) and taking into account for the first relation in (3). We use the Laplace transform (12) and obtain the Laplace image $u^{L}(x,p)$ of the vertical displacements

(14) $$u^{L}(x,p)=\frac{1+v}{1-v}\alpha \frac{1}{\sqrt{\frac{p}{\kappa }}}\left(A_{1}\mathrm{ch}\sqrt{\frac{p}{\kappa }}x+A_{2}\mathrm{sh}\sqrt{\frac{p}{\kappa }}x\right)-A_{3}B(x)+A_{4}$$

where $A_{1}$, $A_{2}$ are from (13) and $A_{3}$, $A_{4}$ are additional arbitrary constants. The function B(x) is defined through μ(x) as follows

(15) $$B(x)=\int_{0}^{x}\frac{d\xi }{\mu (\xi )},\;0\leq x\leq h$$

where μ(x) is a continuous function and does not vanish μ(x)≠0 for any x∈[0,h].

To determine constants $A_{k}$, k=1−4 in (13), (14) we apply the Laplace transform to boundary conditions (4), (6), (9) and (10), which results in

(16) $$u^{L}(h,p)=-\Delta^{L}(p)+u_{w}^{L}(p)$$

(17) $$u^{L}(0,p)=0$$

(18) $$K\frac{dT^{L}(h,p)}{dx}=-fV\sigma_{xx}^{L}(h,p)$$

(19) $$K\frac{dT^{L}(0,p)}{dx}=kT^{L}(0,p)$$

where

(20) $$u_{w}^{L}(p)=-fVK^{*}\frac{\sigma_{xx}^{L}(h,p)}{p}$$

(21) $$\sigma_{xx}^{L}(x,p)=\frac{2(1-v)}{1-2v}\mu_{1}\left(\frac{du^{L}(x,p)}{dx}-\frac{1+v}{1-v}\alpha T^{L}(x,p)\right)$$

where $\mu_{1}=\mu (h)$ is the value of the shear modulus at the upper coating boundary and $\Delta^{L}(p)$ is the Laplace image of the function Δ(t), representing half-plane *I* displacement towards the coating.

Substituting (13), (14), (20), (21) into boundary conditions (16)–(19), we obtain the linear system for the determination of the constants $A_{k}$, k=1−4. Solving this system, we obtain the Laplace images of the temperature, displacement and stress distribution in the coating as follows

(22) $$T^{L}(x,p)=\frac{1-\nu }{1+\nu }\frac{\hat{V}}{\alpha h}\Delta^{L}(p)\frac{hB^{\prime }(h)}{B(h)}\frac{N_{T}(x,z)}{R(z)}$$

(23) $$N_{T}(x,z)=\sqrt{z}\left(\mathrm{Bi}\mathrm{sh}\sqrt{z}\frac{x}{h}+\sqrt{z}\mathrm{ch}\sqrt{z}\frac{x}{h}\right)$$

(24) $$u^{L}(x,p)=-\Delta^{L}(p)\cdot \frac{N_{u}^{0}(x,z)}{R(z)}$$

(25) $$N_{u}^{0}(x,z)=zr(h,z)\frac{B(x)}{B(h)}-\hat{V}\frac{hB^{\prime }(h)}{B(h)}\left(r(x,z)-\mathrm{Bi}\right)$$

(26) $$\sigma_{xx}^{L}(x,p)=\frac{2(1-v)}{1-2v}\mu (x)\Delta^{L}(p)\frac{hB^{\prime }(x)}{B(h)}\cdot \frac{N_{\sigma }^{0}(z)}{R(z)}$$

(27) $$N_{\sigma }^{0}(z)=zr(h,z)$$

where

(28) $$R(z)=zr(h,z)-\hat{V}\eta \left(\left(1-k_{w}\right)r(h,z)-\mathrm{Bi}\right)$$

(29) $$r(x,z)=\mathrm{Bi}\;\mathrm{ch}\sqrt{z}\frac{x}{h}+\sqrt{z}\mathrm{sh}\sqrt{z}\frac{x}{h}$$

$$z=\frac{p}{\kappa }h^{2},\;\mathrm{Bi}=\frac{kh}{K},\;k_{w}=\frac{1-v}{1+v}\frac{KK^{*}}{\alpha \kappa },\;\hat{V}=\frac{fV\alpha }{K}\frac{2\mu_{1}(1+v)h}{1-2v}.$$

For the image $u_{w}^{L}(p)$ of the coating wear, we obtain

(30) $$u_{w}^{L}(p)=k_{w}\hat{V}\frac{hB^{\prime }(h)}{B(h)}\Delta^{L}(p)\frac{r(h,z)}{R(z)}$$

We apply the inverse Laplace transform to the Laplace images $T^{L}(x,p)$, $u^{L}(x,p)$, $\sigma_{xx}^{L}(x,p)$ and obtain the problem solution in the convolution form (t>0)

(31) $$T(x,t)=\frac{1-v}{1+v}\frac{\hat{V}}{\alpha h}\cdot \frac{hB^{\prime }(h)}{B(h)}\int_{0}^{t}\Delta (\tau )f_{T}(x,t-\tau )d\tau$$

(32) $$f_{T}(x,t)=\frac{1}{2\pi i}\int_{\Gamma }\frac{N_{T}(x,z)}{t_{\kappa }R(z)}e^{z\tilde{t}}dz$$

(33) $$u(x,t)=-\int_{0}^{t}\Delta (\tau )f_{u}^{0}(x,t-\tau )d\tau$$

(34) $$f_{u}^{0}(x,t)=\frac{1}{2\pi i}\int_{\Gamma }\frac{N_{u}^{0}(x,z)}{t_{\kappa }R(z)}e^{z\tilde{t}}dz$$

(35) $$\sigma_{xx}(x,t)=-\frac{2(1-v)}{(1-2v)B\left(h\right)}\int_{0}^{t}\Delta (\tau )f_{\sigma }^{0}(x,t-\tau )d\tau$$

(36) $$f_{\sigma }^{0}(x,t)=\frac{1}{2\pi i}\int_{\Gamma }\frac{N_{\sigma }(x,z)}{t_{\kappa }R(z)}e^{z\tilde{t}}dz$$

where $\tilde{t}=\frac{t}{t_{\kappa }}$, $t_{\kappa }=\frac{h^{2}}{\kappa }$.

To obtain (35) from (26) we take into account that $\mu (x)B^{\prime }(x)\equiv 1$. Coating surface wear $u_{w}(t)$ is determined by inverting $u_{w}^{L}(p)$ in (30)

(37) $$u_{w}(t)=k_{w}\hat{V}\frac{hB^{\prime }(h)}{B(h)}\int_{0}^{t}\Delta (\tau )f_{w}(x,t-\tau )d\tau$$

(38) $$f_{w}(x,t)=\frac{1}{2\pi i}\int_{\Gamma }\frac{r(h,z)}{t_{\kappa }R(z)}e^{z\tilde{t}}dz$$

To find out the existence conditions of integrals in (32), (34), (36), (38) we analyze integrands for 0≤x≤h for large values of the integration variable z (argz=π/2):

(39) $$N_{T}(x,z)R^{-1}(z)=O\left(z^{-1/2}\right)\;|z|\to \infty \;N_{u}^{0}(x,z)R^{-1}(z)=\frac{B(x)}{B(h)}+O\left(z^{-1/2}\right)\;|z|\to \infty \;N_{\sigma }^{0}(x,z)R^{-1}(z)=1+O\left(z^{-1/2}\right)\;|z|\to \infty \;r(h,z)R^{-1}(z)=O\left(z^{-1}\right)\;|z|\to \infty$$

From (39) it follows that the integrands in (34), (36) do not decay at infinity (at |z|→∞), and the corresponding integrals are divergent and understood in the generalized sense [50]. After regularization of integrals (34), (36) and separation of the generalized part, expressions for the displacements u(x,t) and stresses $\sigma_{xx}(x,t)$ can be written as follows (t>0)

(40) $$u(x,t)=-\frac{B(x)}{B(h)}\Delta (t)-\int_{0}^{t}\Delta (\tau )f_{u}(x,t-\tau )d\tau \;0\leq x\leq h,\;t>0$$

(41) $$f_{u}(x,t)=\frac{1}{2\pi i}\int_{\Gamma }\frac{N_{u}(x,z)}{t_{\kappa }R(z)}e^{z\tilde{t}}dz$$

(42) $$N_{u}(x,z)=N_{u}^{0}(x,z)-\frac{B(x)}{B(h)}R(z)$$

(43) $$\sigma_{xx}(x,t)=-\frac{2(1-v)}{\left(1-2v)B(h\right)}\left(\Delta (t)-\int_{0}^{t}\Delta (\tau )f_{\sigma }(x,t-\tau )d\tau \right)\;0\leq x\leq h,t>0$$

(44) $$f_{\sigma }(x,t)=\frac{1}{2\pi i}\int_{\Gamma }\frac{N_{\sigma }(x,z)}{t_{\kappa }R(z)}e^{z\tilde{t}}dz$$

(45) $$N_{\sigma }(x,z)=N_{\sigma }^{0}(x,z)-R(z)$$

where $\tilde{t}$ and $t_{\kappa }$ are previously identified, and the contour of integration $\Gamma =\left\{z:-i\infty +dt_{\kappa },+i\infty +dt_{\kappa }\right\}$ is a straight line in the complex plane of integration variable z, which is parallel to the imaginary axis and a distance from equal to the value $dt_{\kappa }$. The value of d is chosen so that the contour of integration passes to the right of all of the isolated singular points of integrands.

Integrands in Equations (40) and (43) at 0≤x≤h are meromorphic functions and decay at large z (−π<argz<π)

(46) $$N_{u}(x,z)R^{-1}(z)=O\left(z^{-1/2}\right)|z|\to \infty \;N_{\sigma }(x,z)R^{-1}(z)=O\left(z^{-1/2}\right)|z|\to \infty$$

These properties of integrands allow us to apply the methods of complex analysis for calculation of the integrals and stability analysis. In quadratures (32), (38), a regularization is carried out to obtain integrands decreasing at infinity, after which they are investigated by the same methods as in (40), (43).

## 4. Poles of the Integrands 

To investigate the stability of the solutions obtained in the previous subsection, we need to study effect of the problem parameters on the integrand poles in integrals (32), (38), (41), (44). The poles of the integrands are zeros of the equation

(47) $$R(z)=zr(h,z)-\eta \hat{V}\left(\left(1-k_{w}\right)r(h,z)-\mathrm{Bi}\right)=0,\;|\arg z|<\pi ,\;|z|<\infty$$

in the complex plane z=ξ+iη, where R(z) and r(x,z) at x=h are from (28) and (29), $\eta =hB^{\prime }(h)/B(h)$ is the coefficient of the FGIM strip. Equation (47) contains four dimensionless parameters of the problem ($k_{w}$, $\hat{V}$, Bi, η), which itself contains dimensional parameters, described after (29). In this case, the dimensionless parameter η, the numerical value of which characterizes the FGM coating, consists of $B^{\prime }(h)=\mu^{-1}(h)$ and B(h) from (15). From the mean value theorem, it follows for B(h) that there is a point c∈[0,h] satisfying the equation

(48) $$B(h)=\mu^{-1}(c)h,\;c\in [0,h]$$

Then, the parameter η can be represented as the averaged value of the shear modulus in the coating μ(c)c∈[0,h] divided by the value of μ(h) at the upper boundary of the coating

(49) $$\eta =\frac{\mu (c)}{\mu (h)},\;c\in [0,h]$$

which is its mechanical meaning.

Locating zeros $\zeta_{k}$ of Equation (47) $R(\zeta_{k})=0$, k=0,1,2,…, their movements in the complex plane ζ=ξ+iη depending on the change in the dimensionless parameters of the problem is the main goal of solving Equation (47).

Equation (47) is solved using numerical methods and complex analysis methods [51]. Analysis of R(z) (47) zeros is similar to [52,53,54] and is performed for $\hat{V}\in [0,\infty )$ at fixed values of dimensionless parameters $k_{w}$, η, Bi. Assuming $\hat{V}=0$, we find the following equation

(50) $$zr(h,z)=z(\mathrm{Bi}\mathrm{ch}\sqrt{z}+\sqrt{z}\mathrm{sh}\sqrt{z})=0$$

to determine the initial estimates $\zeta_{k}^{0}=\zeta_{k}(0)$, k=0,1,2,… of zeros $\zeta_{k}(\hat{V})$, k=0,1,2,… of Equation (47). In general case, Equation (50) does not allow convenient analytic solutions. However, at Bi=0 Equation (50) possesses analytic solution, and the initial estimations $\zeta_{k}^{0}$, k=0,1,2,… of zeros $\zeta_{k}$, k=0,1,2,… of Equation (47) are found from

(51) $$\zeta_{k}^{0}=-{\left(\pi k\right)}^{2},\;k=0,1,2,\ldots$$

And do not depend on $k_{w}$, $\hat{T}$. At Bi=∞, from (50) we obtain another initial estimations $\zeta_{k}^{0}$, k=0,1,2,… as follows

(52) $$\zeta_{k}^{0}=-\pi^{2}{\left(k+1/2\right)}^{2},\;k=0,1,2,\ldots$$

Asymptotic estimation of $\zeta_{k}^{0}$ for large numbers of k has the form of (51). From Formulas (51) and (52) it follows that the initial estimations $\zeta_{k}^{0}$, k=0,1,2,… of function R(z) of zeros from (47) are located on the negative part of the real axis or at the origin. As $\hat{V}$ changes from 0 to ∞ at fixed $k_{w}$, Bi, η the first two poles $\zeta_{0}$ and $\zeta_{1}$ can be located: I—on the real axis Re(ζ0,ζ1)<0, Im(ζ0,ζ1)=0 at $0<\hat{V}<{\hat{V}}_{I}$; II—in the vertical strip l−<Re(ζ0,ζ1)<0, |Im(ζ0,ζ1)|<∞ at ${\hat{V}}_{I}<\hat{V}<{\hat{V}}_{\mathrm{II}}$; III—in the vertical strip 0<Re(ζ0,ζ1)<l+, |Im(ζ0,ζ1)|<∞ at ${\hat{V}}_{\mathrm{II}}<\hat{V}<{\hat{V}}_{\mathrm{III}}$; IV—on the positive part of the real axis Re(ζ0,ζ1)>0, Im(ζ0,ζ1)=0 at ${\hat{V}}_{\mathrm{III}}<\hat{V}<\infty$. 

**Figure 2:**
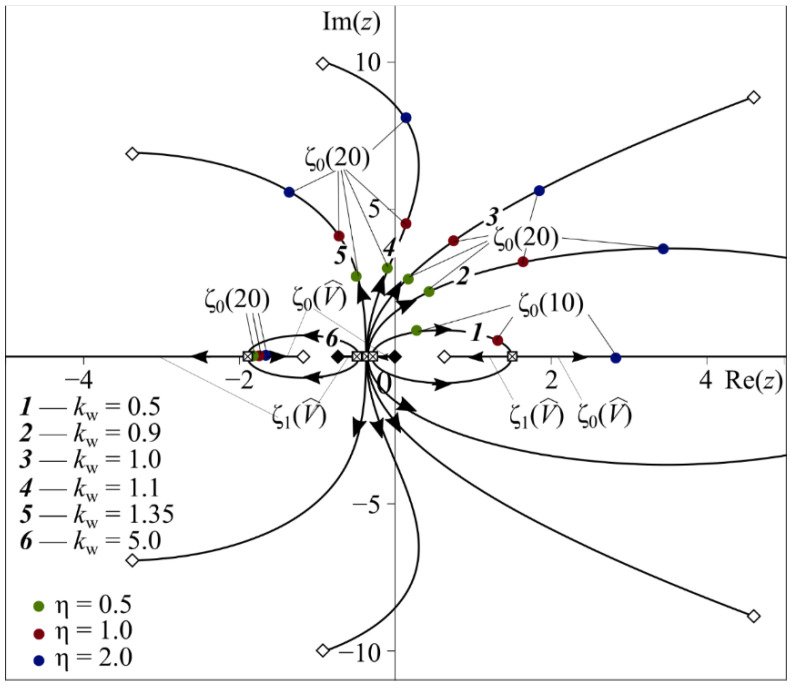
Movement of zeros of R(z) (47) in the complex plane when $\hat{V}$ changes from 0 to ∞.

The numbers I, II denote regions (we will call them domains of stability), where Re(ζ0,ζ1)<0 at $0<\hat{V}<{\hat{V}}_{\mathrm{II}}$, while III, IV denote the regions (we will call them domains of instability), where Re(ζ0,ζ1)>0 at ${\hat{V}}_{\mathrm{II}}<\hat{V}<\infty$. Figure 2 shows some examples of the poles $\zeta_{0}\left(\hat{V}\right)$ and $\zeta_{1}\left(\hat{V}\right)$ trajectories when $\hat{V}$ changes from 0 to ∞ at fixed Bi = 100 for three different values of η = 0.5, 1.0, 2.0 (green, red, blue colors) and following values of $k_{w}$= 0.5 (curve ***1***), 0.9 (***2***), 1.0 (***3***), 1.1 (***4***), 1.35 (***5***), 5.0 (***6***). Solid diamonds indicate locations of $\zeta_{0}\left(\hat{V}\right)$ and $\zeta_{1}\left(\hat{V}\right)$ at $\hat{V}$= 0, while empty ones correspond to $\hat{V}\to \infty$. The point of the trajectory marked as a crossed-out square is a point, after crossing which, with increasing $\hat{V}$, the real poles $\zeta_{0}\left(\hat{V}\right)$ and $\zeta_{1}\left(\hat{V}\right)$ become a pair of complex conjugated poles, and vice versa. Note that even small change in the parameter $k_{w}$, which is proportional to the ratio of the wear factor $K^{*}$ and the thermal expansion coefficient α, leads to significant variation of $\zeta_{0}\left(\hat{V}\right)$ and $\zeta_{1}\left(\hat{V}\right)$ trajectories and, to a lesser extent, trajectories of other $\zeta_{k}\left(\hat{V}\right)$, k=2,3,4,…. If wear is extensive ($k_{w}>1$), both $\zeta_{0}$, $\zeta_{1}$ and all others $\zeta_{k}$, k=2,3,4,⋯ are located in regions I, II (curves ***4***–***6*** on Figure 2). When thermal expansion of the coating material prevails over wear ($0<k_{w}<1$), the poles $\zeta_{0}$ and $\zeta_{1}$ move to the right complex half-plane to regions III, IV (curves ***1***–***3*** on Figure 2).

One significant property of the poles from regions II, III is noted; they are complex conjugated, i.e., $\zeta_{1}=\bar{\zeta_{0}}$ and $\zeta_{0}=\bar{\zeta_{1}}$. 

Wear causes a dramatic effect on the poles’ behavior. In quasi-static sliding contact problems with frictional heating but without wear, the poles $\zeta_{0}$ and $\zeta_{1}$ remain on the real axis for any $\hat{V}\in \left[0,\infty \right)$ [52,53]. In corresponding problems accounting for wear, the poles $\zeta_{0}$ and $\zeta_{1}$ can have a non-zero imaginary part, and in this case become complex conjugates $\zeta_{1}=\bar{\zeta_{0}}$, $\zeta_{0}=\bar{\zeta_{1}}$.

## 5. Formulas for Exact Solution

When the poles $\zeta_{k}$, k=0,1,2,… are known, to calculate the integrals in (32), (41), (44) we calculate the sum of the residues at the points $z=\zeta_{k}$. For all simple poles we obtain

(53) $$\frac{1}{2\pi i}\int_{\Gamma }\frac{N_{a}(x,z)}{t_{\kappa }R(z)}e^{z\tilde{t}}dz=\sum_{k=0}^{\infty }C_{a}\left(x,\zeta_{k}\right)e^{\zeta_{k}\tilde{t}},$$

(54) $$C_{a}(x,z)=\frac{N_{a}(x,z)}{t_{\kappa }R^{\prime }(z)}$$

By replacing symbolic index a in (53), (54) with T, u or σ, Equation (53) can be used to calculate integral in (32), (41), (44), respectively. If $\zeta_{k}$ and $\zeta_{k+1}$, k=0,1,2,… are complex conjugates ($\zeta_{k+1}=\bar{\zeta_{k}}$), then

(55) Ca(x,z)ezt˜=2ReNa(x,z)tκR′(z)ezt˜

and summation in (53) can be entered by even numbers k=2n, n=0,1,2,… for complex conjugate $\zeta_{k}$, k=0,1,2,…. Using (54) for (32), (41), (44) we obtain

(56) $$f_{a}(x,t)=\frac{1}{2\pi i}\int_{\Gamma }\frac{N_{a}(x,z)}{t_{\kappa }R(z)}e^{z\tilde{t}}dz=\sum_{k=0}^{\infty }C_{a}\left(x,\zeta_{k}\right)e^{\zeta_{k}\tilde{t}}a=T,u,\sigma$$

and write out the problem solution in form of series

(57) $$T(x,t)=\frac{1-v}{1+v}\frac{\hat{V}}{\alpha }\frac{B^{\prime }(h)}{B(h)}\sum_{k=0}^{\infty }C_{T}\left(x,\zeta_{k}\right)D\left(\zeta_{k},t\right)\;0\leq x\leq h,\;t>0$$

(58) $$u(x,t)=-\frac{B(x)}{B(h)}\Delta (t)+\sum_{k=0}^{\infty }C_{u}\left(x,\zeta_{k}\right)D\left(\zeta_{k},t\right)\;0\leq x\leq h,\;t>0$$

(59) $$\sigma_{xx}(x,t)=-\frac{2(1-v)}{\left(1-2v)B(h\right)}\left(\Delta (t)-\sum_{k=0}^{\infty }C_{\sigma }\left(x,\zeta_{k}\right)D\left(\zeta_{k},t\right)\right)\;0\leq x\leq h,\;t>0$$

where $C_{a}(x,z)$ is calculated either by (54) or (55), and D(z,t) is calculated as follows

(60) $$D(z,t)=\int_{0}^{t}\Delta (\tau )\exp\left(z(t-\tau )/t_{\kappa }\right)d\tau \;t>0$$

Calculating $f_{w}^{0}(t)$ in (38) by relation

(61) $$f_{w}^{0}(t)=\frac{1}{2\pi i}\int_{\Gamma }\frac{r(h,z)}{t_{\kappa }R(z)}e^{z\tilde{t}}dz=\sum_{k=0}^{\infty }B_{w}\left(\zeta_{k}\right)e^{\zeta_{k}\tilde{t}},\;C_{w}=\frac{r(h,z)}{t_{\kappa }R^{\prime }(z)}$$

and substituting it into (37), we obtain a formula for calculation of the coating wear

(62) $$u_{w}(t)=k_{w}\hat{V}\frac{hB^{\prime }(h)}{B(h)}\sum_{k=0}^{\infty }C_{w}\left(\zeta_{k}\right)D(\zeta_{k},t),\;t>0$$

The horizontal displacements w(x,t) are determined from (1), (5), (7) and after integrating (1) take the form

(63) $$w(x,t)=-fB(x)\sigma_{xx}(h,t)\;0\leq x\leq h,\;t>0$$

## 6. Domains of Stability and Instability

Analysis of Formulas (57)–(59) for T(x,t), u(x,t), $\sigma_{xx}(x,t)$ shows that, in case of Re(ζk)<0, k=0,1,2,… the solution is stable and tends towards a stationary state with increasing t. However, if at least one of $\zeta_{k}$, k=0,1,2,… has Re(ζk)>0, then the magnitude of the solution grows indefinitely when t→∞ and oscillates with the frequency Im(ζk)≠0, which indicates instability of the problem. If the indentation law Δ(t) is a bounded function

m<Δ(t)<M m,M>0, 0<t<∞

then the following estimate for the integral in (60) takes place

|D(ζk,t)|≥m|1−eζkt˜ζk| at Re(ζk)>0 k=0,1,2,…

Trajectories of the poles $\zeta_{k}\left(\hat{V}\right)$, k=0,1,2,…$\hat{V}\in \left[0,\infty \right)$, lying in the left complex half-plane (Re(ζk)<0), correspond to the stable solution, and therefore we refer to domains I, II as the domains of stable solutions. Domains III, IV, lying in the right complex half-plane (Re(ζk)>0, k=0,1), can be referred to as the domains of unstable solutions, because in domain III $\underset{t\to \infty }{\lim}T(h,t)$ and $\underset{t\to \infty }{\lim}\sigma_{xx}(h,t)$ do not exist (because Im(ζk)≠0, k=0,1), and in domain IV we have $\underset{t\to \infty }{\lim}T(h,t)=\underset{t\to \infty }{\lim}\sigma (h,t)=\infty$ (because Im(ζk)=0, k=0,1).

**Figure 3:**
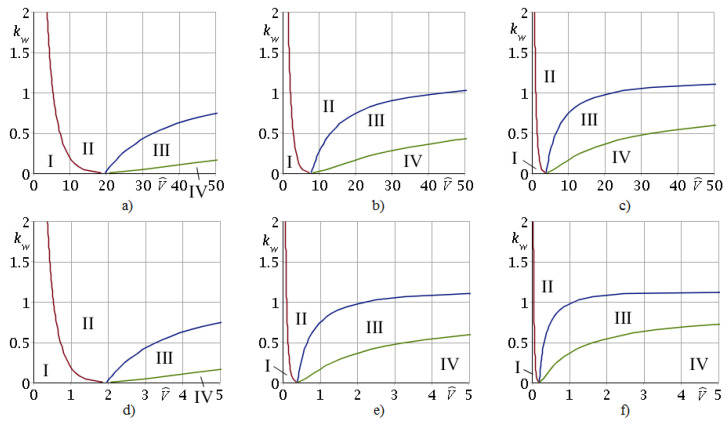
Domains of stability (I, II) and instability (III, IV) of the problem solution at Bi = 100 for different values of η: (**a**) 0.1, (**b**) 0.25, (**c**) 0.5, (**d**) 1.0, (**e**) 5.0, (**f**) 10.0.

Figure 3 presents domains of stability (I, II) and instability (III, IV) in plane ($\hat{V}$, $k_{w}$) and boundaries between them for Bi = 100 and different values of η = 0.1; 0.25; 0.5; 1.0; 5.0; 10.0. These plots show that, at fixed $k_{w}$, $\hat{V}$, η the parameter Bi affects the position of the boundaries of stability and instability. The point of intersection of boundaries I–IV, lying on axis $\hat{V}$ (Figure 3), shifts depending on Bi and has a coordinate ${\hat{V}}_{*}=2\mathrm{Bi}{\left(2+\mathrm{Bi}\right)}^{-1}\eta^{-1}$ ($k_{w}=0$). At η=1 this result coincides with [41]. The formula for ${\hat{V}}_{*}$ shows the effect of the parameter η (Figure 3) on the boundaries of domains I–IV.

**Figure 4:**
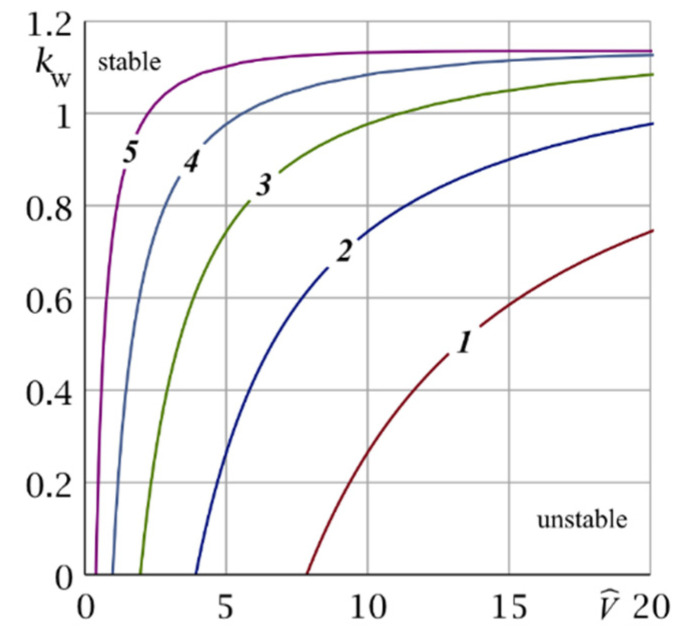
Boundaries between stability and instability domains of the problem solution in the ($\hat{V}$, $k_{w}$) plane at Bi = 100 for different values of η: ***1***—0.25, ***2***—0.5, ***3***—1.0, ***4***—2.0, ***5***—5.0.

Note that the dimensionless parameter η, which characterizes inhomogeneity of the FGM of the coating, significantly affects the boundary between domains of stable (II) and unstable (III) solutions for any $k_{w}$. These boundaries are presented in Figure 4 in detail. One can observe that with decrease in η, the stable solution region increases.

## 7. Features of Wear of FGM Coating 

In Section 5 we obtained the exact formulas for the main characteristics of the problem: temperature T(x,t) (57), displacements u(x,t) (58), stresses $\sigma_{xx}(x,t)$ (59) and coating wear $u_{w}(t)$ (62) on sliding contact. These functions depend on the shear modulus μ(x) variation by the coating depth 0≤x≤h. To illustrate the solution, we assume that the shear modulus μ(x) varies according to a power (parabolic) law

(64) $$\mu (x)=\mu_{0}\left(a{\left(\frac{x}{h}\right)}^{2}+b\frac{x}{h}+c\right)$$

$$a=2\left(\frac{\mu_{1}}{\mu_{0}}-2\frac{\mu_{1/2}}{\mu_{0}}+1\right),\;b=-\left(\frac{\mu_{1}}{\mu_{0}}-4\frac{\mu_{1/2}}{\mu_{0}}+3\right),\;c=1$$

where $\mu_{0}=\mu (0)$, $\mu_{1}=\mu (h)$, $\mu_{1/2}=\mu (h/2)$. This means that the shear modulus is equal to $\mu_{0}$ at the coating–substrate interface (x=0), to $\mu_{1}$ at the contact interface (x=h), and to $\mu_{1/2}$ in the middle of the coating. If $\mu_{1/2}=\left(\mu_{1}+\mu_{0}\right)/2$ then a=0 and the law (64) becomes linear.

Calculating integral (15) of the function in (64), we obtain the expression for B(x)

(65) $$B(x)=\frac{h}{\mu_{1}}\{\begin{array}{cc} \frac{2\chi }{\sqrt{-D}}\mathrm{arctg}\frac{\sqrt{-D}}{\theta (x)} & D<0\\ \frac{\chi }{\sqrt{D}}\ln\left|\frac{1+\theta_{-}(x)}{1-\theta_{+}(x)}\right| & D>0\\ -4\frac{c}{b}\frac{\chi }{\theta (x)} & D=0 \end{array}$$

where $D=b^{2}-4ac$, $\chi =\frac{\mu_{1}}{\mu_{0}}$, $\theta (x)=2c+b\frac{x}{h}$, $\theta_{\pm }(x)=\frac{2a}{b\pm \sqrt{D}}\cdot \frac{x}{h}$.

Equation (65) allows one to determine other characteristics depending on μ(x)

(66) $$B^{\prime }(x)=\frac{1}{\mu (x)}=\frac{1}{\mu_{0}\left(a{\left(\frac{x}{h}\right)}^{2}+b\frac{x}{h}+c\right)}$$

(67) $$B^{\prime }(h)=\frac{1}{\mu_{0}\chi }$$

(68) $$B(h)=\frac{h}{\mu_{1}}\{\begin{array}{cc} \frac{2\chi }{\sqrt{-D}}\mathrm{arctg}\frac{\sqrt{-D}}{2c+b} & D<0\\ \frac{\chi }{\sqrt{D}}\ln\left|\frac{1+b_{-}^{1}}{1-b_{+}^{1}}\right| & D>0\\ -4\chi \frac{c}{b(2c+b)} & D=0 \end{array}$$

where $D=b^{2}-4ac$, $b_{\pm }^{1}=\frac{2a}{b\pm \sqrt{D}}$.

Then, from (67), (68) we obtain an expression for the parameter η, which characterizes the inhomogeneity of the FGM coating. In case of the parabolic variation of μ(x) at 0≤x≤h, according to (64), it has the form

(69) $$\eta =\frac{hB^{\prime }(h)}{B(h)}=\frac{h}{\mu_{1}B(h)}$$

The law Δ(t) of indentation of a hard abrasive (half-plane *I*) into the coating is given by the formula

(70) $$\Delta (t)=\Delta_{0}\left(\left(e^{\varepsilon t}-1\right)H(t_{0}-t)+H(t-t_{0})\right),\;t>0$$

where H(t) is the Heaviside step function. Formula (70) assumes that the time section of indentation $0<t\leq t_{0}$ is active and, when $t>t_{0}$ it is passive, since $\Delta (t)=\Delta_{0}$ at $t>t_{0}$. 

The nature of the loss of thermoelastic stability of the main parameters of the contact (temperature T(h,t), contact stresses $\sigma_{xx}(h,t)$, coating wear $u_{w}(t)$) is studied in detail in Section 6 depending on the value of the parameter η. The boundaries of the thermoelastic stability region on the set of parameter values ($\hat{V}$, $k_{w}$) at fixed values η and Bi are also indicated there. 

Let us study the effect of the parameter η of the considered thermoelastic problem on wear by a hard abrasive (half-plane *I*) of an elastic strip made from aluminum with a graded content of alumina (Al_2_O_3_) on the main characteristics of the contact: temperature T(h,t) from (57), contact stresses $p(t)=-\sigma_{xx}(h,t)$ from (59), coating wear $u_{w}(t)$ from (62) and wear rate ${\dot{u}}_{w}(t)$. This FGM strip is characterized by an increased shear modulus $\mu_{1}=\mu (h)$ = 125.0 GPa at the contact and shear modulus at the interface with the substrate $\mu_{0}=\mu (0)$ = 25.0 GPa, ν = 0.34, κ = 88.1 × 10^−6^ mm^2^/s, α = 22.9 × 10^−6^ 1/K, K = 209.3 Bt/(m·K), *f* = 0.47, h = 20 mm, $\Delta_{0}$ = 5 mm, $t_{0}$ = 5 s, *V* = 2.5 mm/s, ε=ln2. We consider three different values $\mu_{1/2}=\mu (h/2)$, which, together with their corresponding values η, are presented in Table 1. 

**Figure 5:**
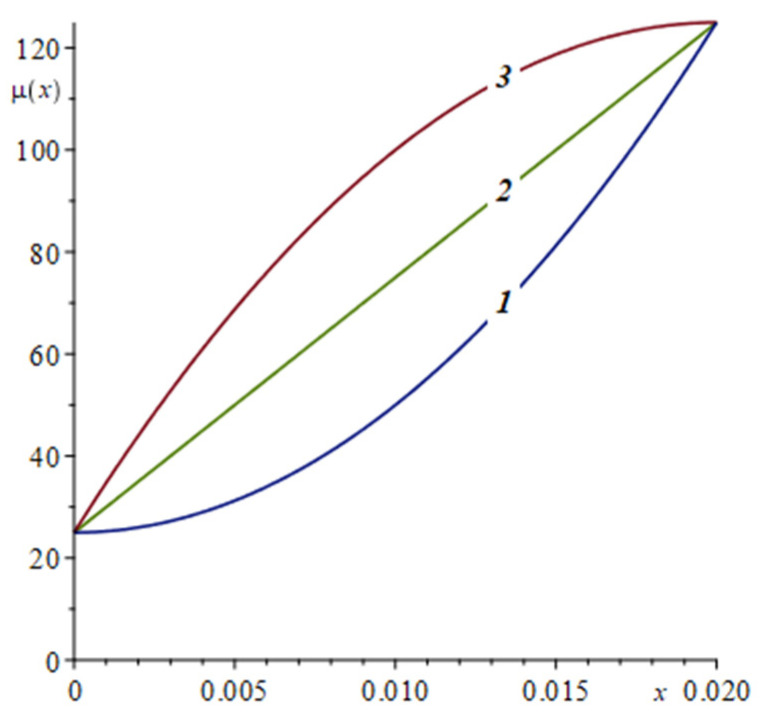
Variation of the shear modulus μ(*x*) by the coating depth at the considered values of $\mu_{1/2}$: ***1***—50, ***2***—75, ***3***—100 GPa.

Variation of the shear modulus μ(x) by the x-coordinate is illustrated in Figure 5.

The wear of the coating surface at the depth $\Delta_{0}$ ends up at $t=t_{w}$, when the coating wear $u_{w}(t)$ equals $\Delta_{0}$, and the contact stress turns to zero ($p(t)=-\sigma_{xx}(h,t)=0$). We call $t_{w}$ the time of the coating wear by amount $\Delta_{0}$. Assuming the wear factor $K^{*}$ = 1.0 × 10^−11^ m^2^/N, we obtain the values of dimensionless parameters $k_{w}$ = 0.511 and Bi=$10^{5}$ using Formula (29). 

Table 2 gives the coating wear time, together with maximum values of contact pressure p(t) and temperature T(h,t) depending on the shear modulus in the middle of the coating $\mu_{1/2}$ and sliding velocity *V*.

**Figure 6:**
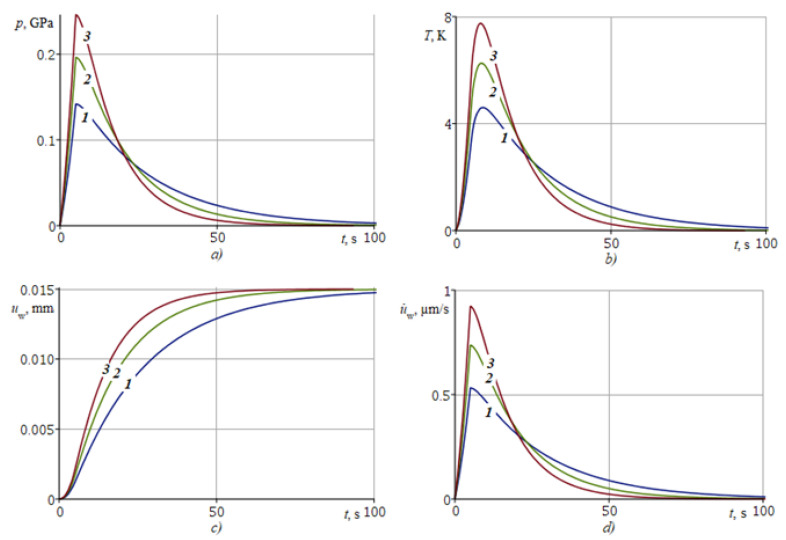
Dependence of the main contact characteristics by time: (**a**) contact pressure $p(t)=-\sigma_{xx}(h,t)$, (**b**) contact temperature T(h,t), (**c**) coating wear $u_{w}(t)$, (**d**) coating wear rate ${\dot{u}}_{w}(t)$, for different values of shear modulus in the middle of the coating $\mu_{1/2}$: ***1***—50, ***2***—75, ***3***—100 GPa.

The effect of the parameter η on the main contact characteristics is illustrated in Figure 6a–d, presenting plots of T(h,t), p(t), $u_{w}(t)$, ${\dot{u}}_{w}(t)$. Curves denoted ***1***, ***2***, ***3*** are plotted for $\mu_{1/2}$ = 50, 75, 100 GPa, respectively.

Figure 6 shows that the coating wear is accompanied by an increase in contact stress p(t) and temperature T(h,t). The growth of the contact stress p(t) is explained by the proportionality of the stresses $\sigma_{xx}\left(x,t\right)$ to the shear modulus μ(x) according to (3). The temperature T(h,t) is proportional to the contact stresses $\sigma_{xx}\left(x,t\right)$, according to (9), and, hence, to the shear modulus μ(x). Since curve ***3*** indicates a greater stiffness of FGM than curve 1 (Figure 5), the values of p(t) and T(h,t) for curve ***3*** will exceed the values of these characteristics for curve 1 (Figure 6). The wear of the coating demonstrating higher stiffness (curve ***3***) will occur faster than the wear of the less stiff coating (curve ***1***), for the same reason according to (8), and also because the vertical movement of abrasive I carried out forcibly according to the law for Δ(t). Hence, it follows that it is possible to experimentally determine the depth-variable shear modulus of the FGM coating under wear conditions in accordance with the kinematic law for Δ(t) of abrasive I, using pressure and temperature sensors at the contact. 

For the preparation of materials with grinding, the power supply of the abrasive is often used due to the contact pressure p(t), which must be kept at a certain level. This restriction makes it possible to calculate the kinematic law for Δ(t) with the condition of the limitation of p(t). Let the formula for the contact stresses be given by the following expression

(71) $$p(t)=p_{0}\left(\left(e^{\delta t}-1\right)H(t_{0}-t)+H(t-t_{0})\right)$$

where $\delta =t_{0}^{-1}\ln2$, and $t_{0}$ characterizes the time of reaching the stationary mode at $t>t_{0}$, at which $p\left(t\right)=p_{0}$. To express Δ(t) in terms of p(t), we use formula (26) in the form

(72) $$p_{}^{L}(p)=-\frac{2(1-v)}{1-2v}\Delta^{L}(p)\frac{h}{B(h)}\cdot \frac{N_{\sigma }^{0}(z)}{R(z)}$$

$\Delta^{L}(p)$ is determined from (72) by the following formula

(73) $$\Delta^{L}(p)=-\frac{1-2v}{2(1-v)}p_{}^{L}(p)\frac{B(h)}{h}\cdot \frac{R(z)}{N_{\sigma }^{0}(z)}$$

After inverting the previous formula, taking into account (27), we obtain the following formula for determining Δ(t) from p(t)

(74) $$\Delta (t)=-\frac{1-2v}{2(1-v)}\frac{B(h)}{h}\int_{0}^{t}p(\tau )\cdot f_{p}\left(t-\tau \right)d\tau$$

(75) $$f_{p}\left(t\right)=\frac{1}{2\pi i}\int_{\Gamma }\frac{R(z)}{t_{\kappa }z\;r(h,z)}e^{z\tilde{t}}dz$$

where $\tilde{t}=\frac{t}{t_{\kappa }}$, $t_{\kappa }=\frac{h^{2}}{\kappa }$, R(z) and r(h,z) are taken from (28), (29), and B(h) is from the expression (15).

The temperature at the contact T(h,t) with limited p(t) will also be limited, and wear is determined according to (62).

Similarly, it is possible to determine Δ(t) so that the temperature is limited, for example, by the following formula

(76) $$T(t)=T_{0}\left(\left(e^{\theta t}-1\right)H(t_{0}-t)+H(t-t_{0})\right)$$

where $\theta =t_{0}^{-1}\ln2$, and $t_{0}$ is the time, when T(t) becomes $T_{0}$ at $t>t_{0}$. Using formula (22) for $T^{L}\left(x,p\right)$ according to the above scheme, we obtain

(77) $$T^{L}(p)=\frac{1-\nu }{1+\nu }\frac{\hat{V}}{\alpha h}\Delta^{L}(p)\frac{hB^{\prime }(h)}{B(h)}\frac{N_{T}(h,z)}{R(z)}$$

$\Delta^{L}(p)$ is determined from (77) by the following formula

(78) $$\Delta^{L}(p)=\frac{1+\nu }{1-\nu }\frac{\alpha h}{\hat{V}}T^{L}(p)\frac{B(h)}{hB^{\prime }(h)}\frac{R(z)}{N_{T}(h,z)}$$

After inverting (78), we obtain

(79) $$\Delta (t)=\frac{1+\nu }{1-\nu }\frac{\alpha h}{\hat{V}}\frac{B(h)}{hB^{\prime }(h)}\int_{0}^{t}T(\tau )\cdot f_{T}\left(t-\tau \right)d\tau$$

(80) $$f_{T}\left(t\right)=\frac{1}{2\pi i}\int_{\Gamma }\frac{R(z)}{t_{\kappa }N_{T}(h,z)}e^{z\tilde{t}}dz$$

where $\tilde{t}=\frac{t}{t_{\kappa }}$, $t_{\kappa }=\frac{h^{2}}{\kappa }$.

When Δ(t) is taken from the expression (79), not only will the temperature on the contact be limited, but also the contact stress p(t). The wear is determined through Δ(t) from (79) according to (62).

## 8. Discussion

The present study was aimed at solving the problem of wear-grinding of a FGM coating with a depth-varying shear modulus. According to the results of the studies presented in the paper, it becomes feasible to solve the inverse problem of determining the law of distribution of the depth-varying shear modulus of the FGM l of the coating. It will be necessary to find out what the minimum set of information is for the unambiguous determination of the shear modulus varying by depth. The solution to this problem is important for the design of FGM coatings with special properties (anti-friction, anti-corrosion, wear-resistant, etc.).

## 9. Conclusions

The considered thermoelastic contact problem of sliding wear by a hard abrasive material at a constant speed over the surface of an elastic coating in the form of a strip made of FGM with an arbitrary shear modulus varying with the depth of the coating made it possible to establish:

− A dimensionless parameter η of the FGIM of the coating, which characterizes the FGM of the coating and its presence in all formulas of the main parameters of the contact with friction;
− Boundaries of the regions of thermoelastic instability in the space of dimensionless parameters of the problem: $k_{w}$, $\hat{V}$, Bi, η;
− Features of the kinematic scheme of wear, which consists in setting an arbitrary law of upsetting of a hard abrasive on the surface of a strip made of FGM and leading to an uncontrolled increase in temperature and contact stresses;
− A special grinding scheme, consisting in the development of such a law of displacement of a hard abrasive into the surface of a strip made of FGM, with the help of which the growth of both the temperature at the contact and the contact stresses is limited.

## Figures and Tables

**Table 1:** Parameters of the shear modulus variation with the coating depth.

Curve No.	$\mu_{1/2},\;\mathrm{GPa}$	η
1	50	0.361287
2	75	0.497067
3	100	0.619564

**Table 2:** Values of main contact characteristics depending on shear modulus in the middle of coating $\mu_{1/2}$ and sliding velocity *V* in case of parabolic variation of the shear modulus μ(x).

$\mu_{1/2},\;\mathrm{GPa}$	*t*_w_, s		$\underset{t\in (0,t_{w}]}{\max}p(t),\;\mathrm{GPa}$		$\underset{t\in (0,t_{w}]}{\max}T(h,t),\;K$	
	*V* = 2.5 mm/s	*V* = 5.0 mm/s	*V* = 2.5 mm/s	*V* = 5.0 mm/s	*V* = 2.5 mm/s	*V* = 5.0 mm/s
50	192.3	124.6	0.142	0.144	4.600	8.996
75	128.9	30.2	0.196	0.201	6.292	12.279
100	93.3	18.6	0.246	0.253	7.749	15.153

## Data Availability

Data are available on request.

## Nomenclature

w(x,t)	horizontal component of displacements in the coating
$\sigma_{xx}(x,t)$	normal component of stresses in the coating
$\sigma_{xy}(x,t)$	shear component of stresses in the coating
T(x,t)	the coating temperature
h	thickness of the coating
V	sliding velocity of the abrasive (half-plane *I*)
μ(x)	shear modulus of the coating material
ν	Poisson’s ratio of the coating material
α	coefficient of linear heat expansion of the coating material
κ	the thermal diffusivity of the coating material
f	the coefficient of friction
$K^{*}$	the proportionality coefficient between the work of friction forces and the volume of removed material
K	the thermal conductivity of the coating material
k	the heat transfer coefficient through the coating–substrate interface
$u_{w}(t)$	coating wear
Q(t)	the amount of frictional heat originated at the contact interface
Δ(t)	the law of indentation of a hard abrasive (half-plane *I*) into the coating
